# Radiofrequency catheter ablation in a patient with dextrocardia, persistent left superior vena cava, and atrioventricular nodal reentrant tachycardia: A case report

**DOI:** 10.1097/MD.0000000000022086

**Published:** 2020-09-04

**Authors:** Zhipeng Zheng, Zhihuan Zeng, Yuliang Zhou, Chichang Li, Wei Zhang

**Affiliations:** a Department of Cardiovascular, The First Affiliated Hospital of Guangdong Pharmaceutical University, Guangzhou; b Dongguan People's Hospital, Dongguan, Guangdong, China.

**Keywords:** dextrocardia, persistent left superior vena cava, radiofrequency catheter ablation, supraventricular tachycardia, venous malformation

## Abstract

**Rationale:**

Dextrocardia is a rare congenital heart disease, while the persistent left superior vena cava (PLSVC) is an uncommon congenital vascular malformation. It is extremely rare for a person to have dextrocardia and PLSVC. A case with a combination of dextrocardia, PLSVC, and atrioventricular nodal reentrant tachycardia has not been reported.

**Patient concerns:**

A 51-year-old woman was admitted to the hospital with palpitations. The physical examination revealed a heart rate of patient increased significantly, and that apex beating was found in the right fifth intercostal space approximately 0.5 cm from the midclavicular line.

**Diagnosis:**

We used different techniques, including electrocardiography, esophagus heart electrophysiology, chest radiograph, and cardiac color Doppler echocardiography to reveal the presence of the combination of dextrocardia, PLSVC, and supraventricular tachycardia.

**Interventions:**

We terminated tachycardia by esophageal pacing and cured patients with radiofrequency catheter ablation (RFCA).

**Outcomes:**

The complex structural anomalies presented great technical challenges for interventional treatments. After consulting the literature, thorough examination and understanding of the structural anatomy and anomalies of the vena cava and cardiac chambers, we successfully treated this patient by RFCA. After half a year of follow-up, the patient did not have palpitations, and no arrhythmia was seen on the electrocardiography.

**Lessons:**

Physicians need to be aware that the key to the success of RFCA, in this case, is to clarify the complexity of the morphological and anatomical structures of dextrocardia accompanying PLSVC and to consult and understand the experience of access vessels reported in relevant cases before the operation.

## Abstract 2

Supplemental Digital Content is available in the text

## 1 Introduction

Dextrocardia is a rare congenital heart disease that is the general term for the heart to move to the right in the chest. It has been reported that the incidence of dextrocardia is 0.1%, which accounts for 0.5% of the cases of adult congenital heart disease.^[1]^ Persistent left superior vena cava (PLSVC) is also called bilateral superior vena cava malformation. Its incidence in the normal population is 0.3% to 0.5%, accounting for 3% to 10% of the cases of congenital heart defects. It is the most common type of systemic venous malformation.^[2,3]^ Supraventricular tachycardia is a common arrhythmia, but it is very rare to have dextrocardia and PLSVC, which may increase the difficulty of radiofrequency catheter ablation (RFCA) due to the variation of anatomical structure position. Here, we report a rare case of a 51-year-old woman with dextrocardia and PLSVC accompanied by supraventricular tachycardia. No similar case has been reported previously. By comparing different vascular pathways of RFCA, we attempted to identify a less damaging and effective treatment. We explore the treatment strategy of dextrocardia with PLSVC and supraventricular tachycardia. The study protocol did not require approval by an ethics review committee.

## 2 Case presentation

**Figure 1:**
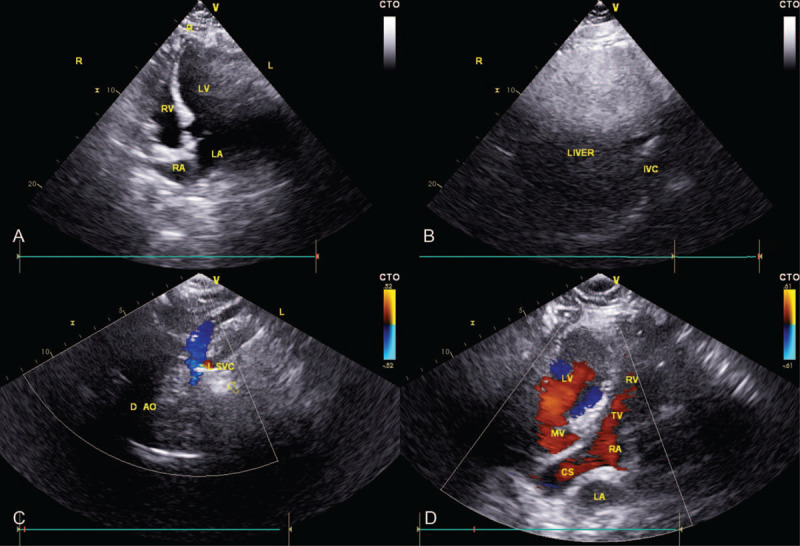
Cardiac color Doppler echocardiography: dextrocardia, live at the same time with the left superior vena cava lead into the right atrium by expanding coronary sinus.

**Figure 2:**
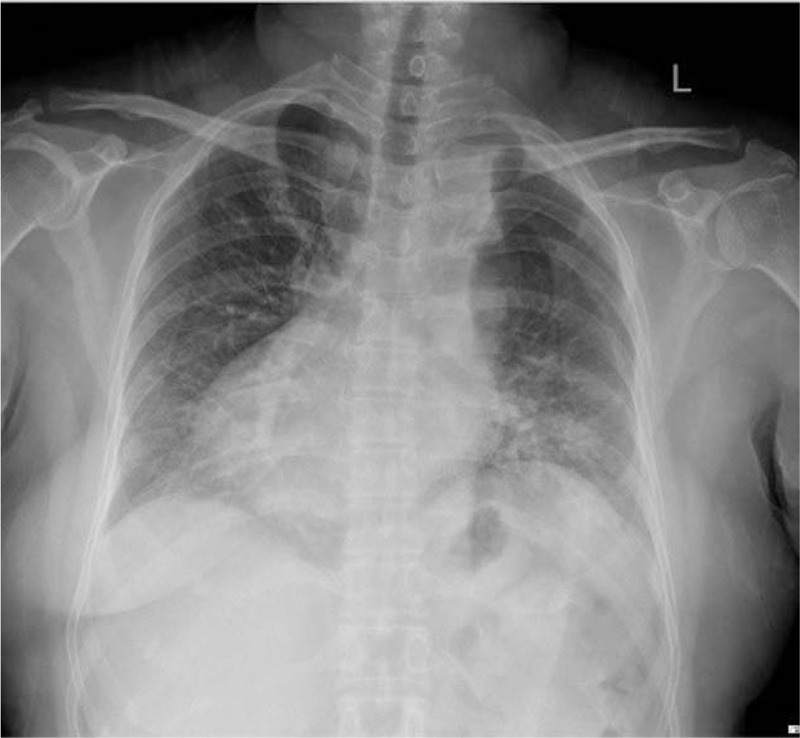
Chest X-ray: dextrocardia.

A 51-year-old female patient was hospitalized for palpitation half a day ago. On admission, the physical examination showed that the blood pressure was 95/78 mm Hg, equal-sized pupils, and a heart rate of 167 bpm, and that apex beating was found in the right fifth intercostal space approximately 0.5 cm from the midclavicular line with no heart murmur. Electrocardiography examination revealed that the heart rate was 152 bpm, the rhythm was regular, there was no P wave, the QRS wave shape and time limit were normal, the R wave of V1-V6 lead decreased gradually (Supplemental Fig. 1). We initially diagnosed the patient as supraventricular tachycardia. Esophagus heart electrophysiology indicated that RP‘ was about 70 ms, and the inhibition of tachycardia could stop the attack of tachycardia, which further confirmed that the patient was supraventricular tachycardia. During the examination of cardiac color Doppler echocardiography, we found that the patient had dextrocardia, and the PLSVC draining blood into the right atrium through the dilated coronary sinus, while the right superior vena cava was absent (Fig. 1). Moreover, chest X-ray also indicates that the patient has dextrocardia (Fig. 2).

**Figure 3:**
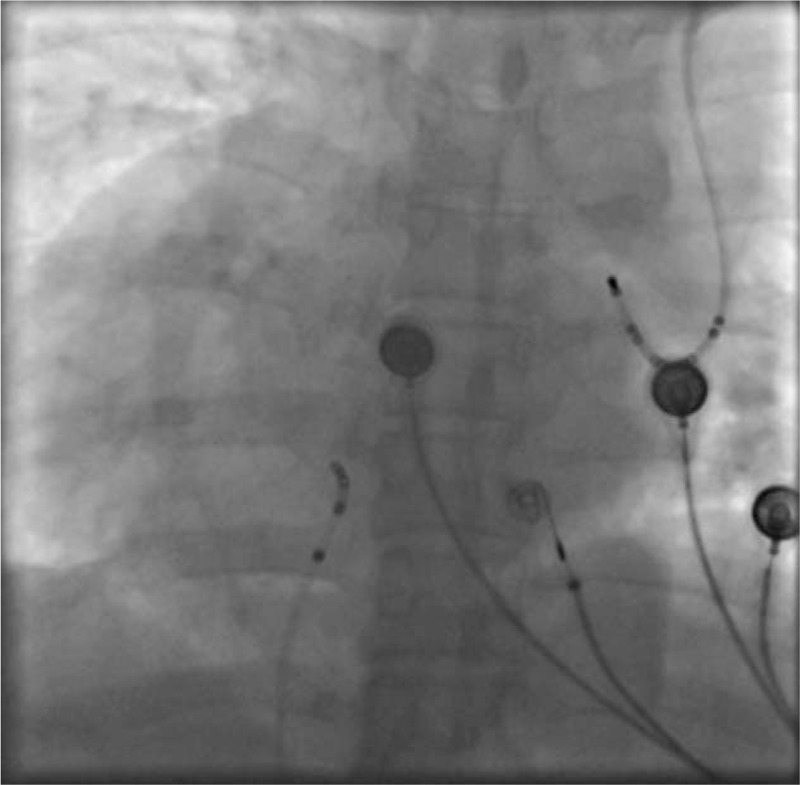
The 10 polar coronary sinus electrodes and the 4 polar mapping electrodes were sent through the sheath.

**Figure 4:**
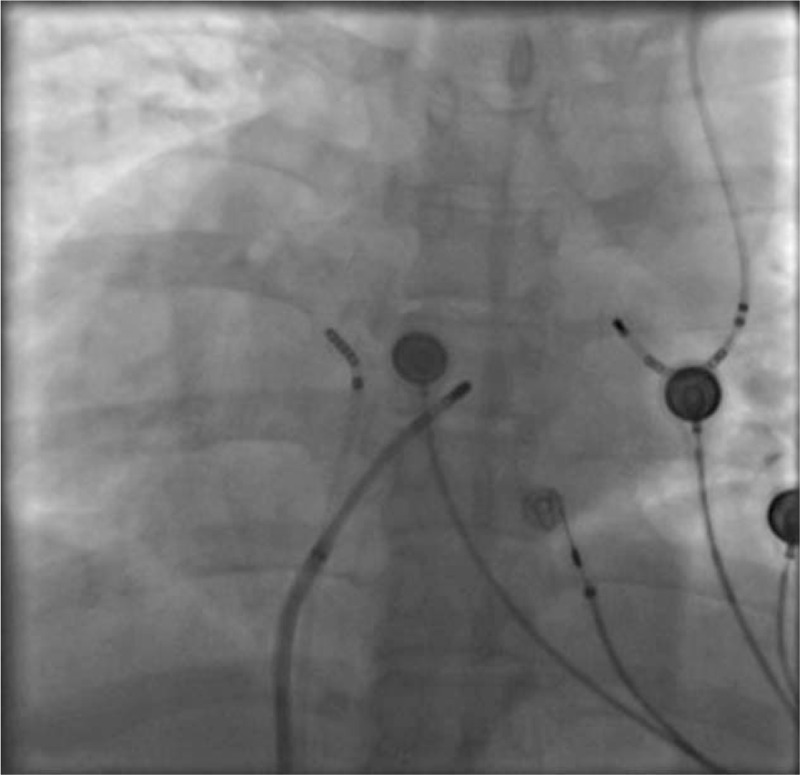
The 8FSR0 sheath was inserted, and then the catheter was put into the large head catheter with temperature control.

**Figure 5:**
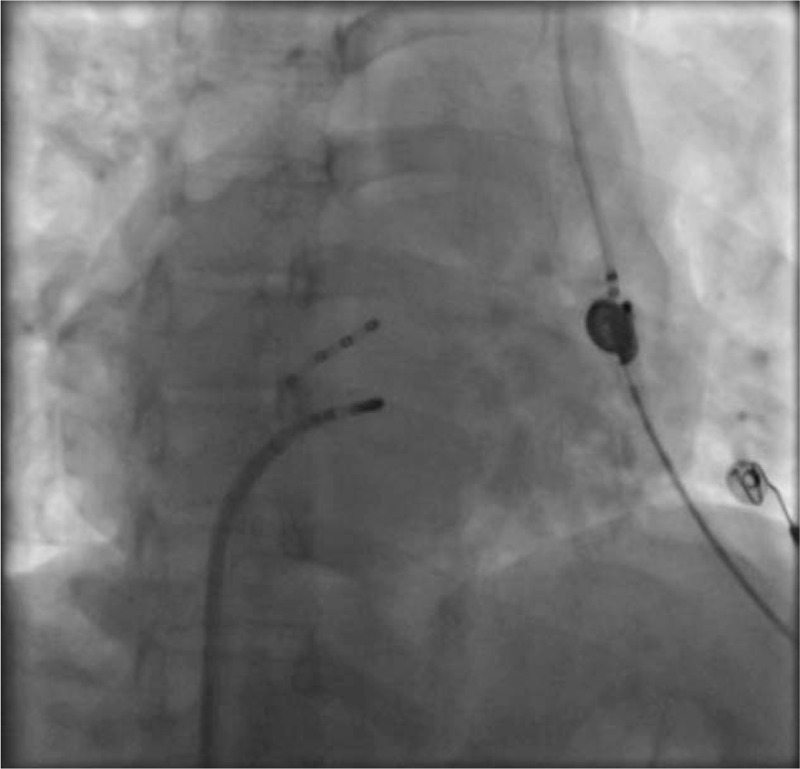
In the left-anterior oblique view of 30°, the target point was detected in the Koch triangle.

Based on the above examinations and clinical manifestations, we diagnosed this patient as having supraventricular tachycardia combined with dextrocardia and PLSVC, and designed the following operation strategy to RFCA. After successful puncture of left subclavian vein and right femoral vein by Seldingers method under local anesthesia, 6F and 7F sheaths were placed respectively, and the 10 polar coronary sinus electrodes and the 4 polar mapping electrodes were sent through the sheath (Fig. 3). When the coronary sinus electrodes were sent, it was again confirmed that the patient had PLSVC static malformation in addition to dextrocardia. Wenckebachs point was stimulated by ventricular S_1_S_1_ for 550ms, and decreased conduction was found in the S_1_S_2_ program scanning, no jumping extension was found, and tachycardia was not induced. Atrial the S_1_S_1_ stimulation of the Wenckebachs point was greater than 300 ms, and no tachycardia attack was induced. The jumping was prolonged in the S_1_S_2_ scan, and no tachycardia attack was induced. After intravenous infusion of isoproterenol to increase heart rate, the tachycardia was induced by rapid stimulation of S_1_S_1_. The intracardiac electrocardiogram showed atrioventricular nodal reentrant tachycardia. After the right femoral vein was punctured again successfully, the 8FSR0 sheath was inserted, and then the catheter was put into the large head catheter with temperature control (Fig. 4). In the left-anterior oblique view of 30°, the target point was detected in the Koch triangle (Fig. 5), showing small A and large V waves, during which there was no H wave. After ablation at 55°C and 30W for several seconds, nodal rhythm and sinus rhythm alternately appeared and then continued to consolidate the ablation for 120 seconds. No prolongation of jumping and no tachycardia attack were found in the repeated stimulation of S_1_S_1_ and S_1_S_2_ in the atrium or in the repeated stimulation of S_1_S_1_ and S_1_S_2_ after intravenous infusion of isoproterenol. It was indicated that the RFCA was successful. The patients returned to the ward safely and continued to be treated with myocardial nutrition and antiplatelet aggregation after the operation.

After half a year of follow-up, the patient did not have palpitations, and no arrhythmia was seen on the electrocardiography (Supplemental Fig. 2).

## 3 Discussion

There are 3 types of dextrocardia based on the position of heart chambers and abdominal organs. The first is true dextrocardia, also known as mirror dextrocardia. Its anatomical structure is completely opposite to that of normal heart, often accompanied by visceral mirror inversion. The second one is cardiac dextroversion, also known as pseudodextrocardia. Although it is located in the right chest and the apex of the heart points to the right, the left atrium, and the left ventricle are still located on the left side, while the right atrium and the right ventricle are still located on the right side. The visceral position of this type is normal, often accompanied by severe congenital malformations of heart vessels and other parts. The third is cardiac disposition, which often causes the heart to move to the right chest due to lung, pleural, or diaphragmatic lesions.^[1,4,5]^ According to the results of color Doppler echocardiography, we report that the patient is cardiac dextroversion, which visceral position is normal, the apex of the heart points to the right, the left atrium and the left ventricle are still on the left, the right atrium and the right ventricle are still on the right, the atrioventricular connection sequence is consistent, and the location of the large vessels is normal. In addition, it is accompanied by a PLSVC vascular malformation.

During fetal development, the left anterior main vein and the left Cuvier tube in the left superior vena cava were not closed in time, resulting in their continued existence after birth and forming a PLSVC.^[6,7]^ There are many types of PLSVC. According to the different drainage sites of left superior vena cava, PLSVC may be classified into 3 types: type I, the left superior vena cava leads into the right atrium through the coronary sinus, which is the most common type,^[8]^ often accompanied by dilation of the coronary sinus; type II, PLSVC drainage into left atrium, commonly accompanied by other more complex congenital cardiovascular malformations; type III, the left superior vena cava is drained into the right superior vena cava through the left innominate vein. In addition, there are some reports that the left superior vena cava can directly flow into the left pulmonary vein.^[9]^ The incidence of arrhythmias in patients with PLSVC is higher since the origin of the sinoatrial node, the atrioventricular node, and the atrioventricular bundle is structurally close to the junction between the main vein and the coronary sinus.^[10]^

This patient is a type I PLSVC combined with cardiac dextroversion and atrioventricular nodal reentrant tachycardia. To our knowledge, this is the first case described in the literature of type I PLSVC with right ventricular transposition and atrioventricular nodal reentrant tachycardia. Only a few cases of supraventricular tachycardia with dextrocardia have been reported. In these references, dextrocardia is almost mirror dextrocardia, and cardiac dextroversion has not been found. When reviewing the relevant literature, we found that cases of the femoral vein or femoral artery as ablation pathway have been reported, but the femoral vein is often used as an ablation pathway.^[11–14]^ In addition, radiofrequency ablation of mirror dextrocardia with Wolff-Parkinson-White syndrome via elbow vein has also been reported.^[15]^ The patient, in this case, is a cardiac dextroversion, which means that the left atrium and left ventricle are still on the left side of the anatomical position of the heart, while the right atrium and right ventricle are still on the right side. Therefore, we used the right femoral vein route to place the 4-pole mapping electrode for ablation. Because of the absence of the right superior vena cava, we used the left subclavian vein to place the coronary sinus electrode.

In this particular case, the main challenge of RFCA is how to locate the ablation target accurately. In addition to the cardiac dextroversion, the patient also has the type I PLSVC. The blood flow of the left superior vena cava is led into the right atrium through the coronary sinus, which makes the coronary sinus expand. The complex anatomical structure of dextrocardia combined with PLSVC may change the location of the ablation target and increase the difficulty of operation. According to the results of the intracardiac electrocardiogram, we determined that the patient was atrioventricular nodal reentrant tachycardia. Considering the abnormal anatomical structure of the patient, we used the left-anterior oblique view of 30°, and detected the target in the Koch triangle area, showing small A and large V waves, and there was no H wave during the period and failed to induce tachycardia after temperature control ablation. The patient recovered well after the operation, and no palpitation occurred in the follow-up.

## 4 Conclusion

We report a rare case of atrioventricular nodal reentrant tachycardia combined with PLSVC and dextrocardia. We successfully completed the RFCA by consulting relevant references and understanding the abnormal nodal anatomy of cardiac dextroversion and PLSVC in detail. By publishing this case, we can share our experience in the diagnosis and treatment of similar cases in this field. It should be pointed out that there is no absolute operation criterion guide when RFCA is used to treat supraventricular tachycardia with dextrocardia and PLSVC. The key to the success of RFCA, in this case, is to clarify the complexity of the morphological and anatomical structures of dextrocardia accompanying PLSVC and to consult and understand the experience of access vessels reported in relevant cases before the operation.

## Acknowledgments

The authors gratefully acknowledge the patient profiled in this report for allowing us to disclose details relating to her case as well as the support from The First Affiliated Hospital of Guangdong Pharmaceutical University.

## Author contributions

**Conceptualization:** Wei Zhang.

**Data curation:** Chichang Li.

**Investigation:** Chichang Li.

**Methodology:** Wei Zhang.

**Resources:** Zhihuan Zeng.

**Validation:** Zhihuan Zeng.

**Visualization:** Yuliang Zhou.

**Writing – original draft:** Zhipeng Zheng.

**Writing – review & editing:** Wei Zhang.

## Supplementary Material

Supplemental Digital Content

## Supplementary Material

Supplemental Digital Content
