# Supplementary figures and images for: Radiofrequency catheter ablation in a patient with dextrocardia, persistent left superior vena cava, and atrioventricular nodal reentrant tachycardia: A case report
Source: Medicine (Baltimore). 2020 Sep 4;99(36):e22086. doi: 10.1097/MD.0000000000022086 (PMC7478451; doi:10.1097/MD.0000000000022086)

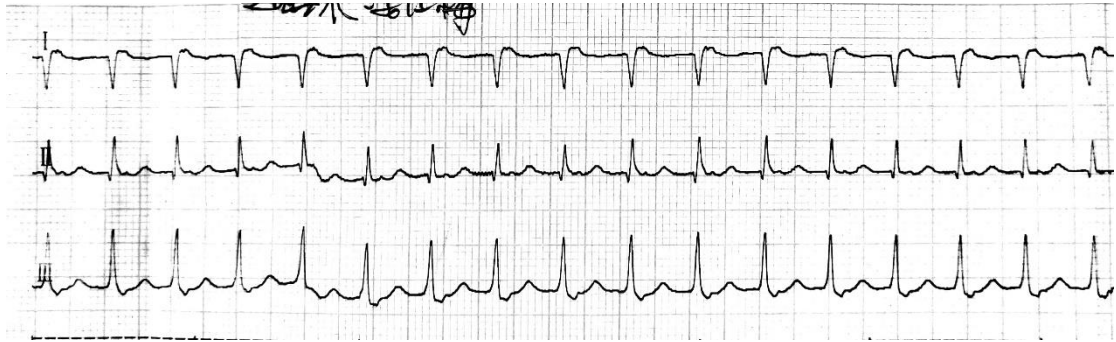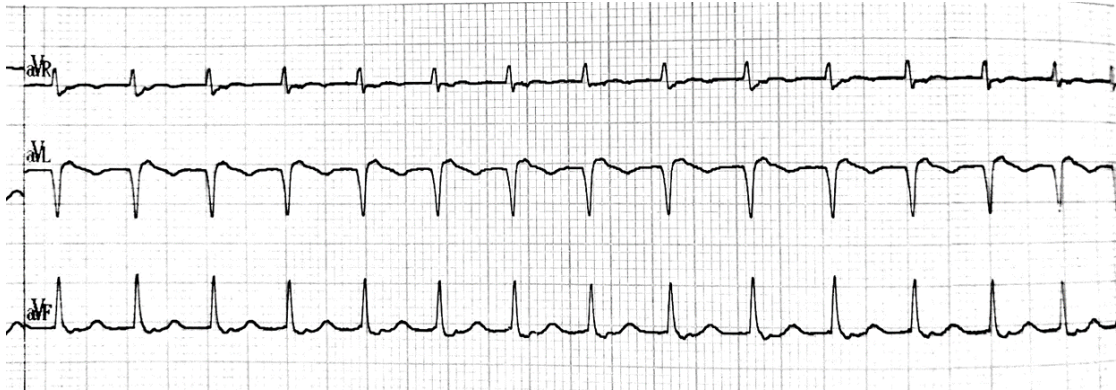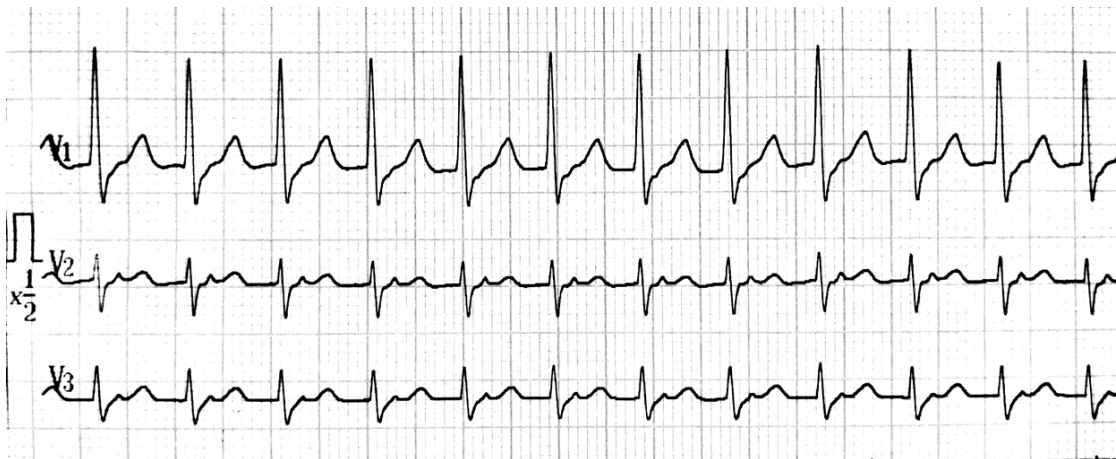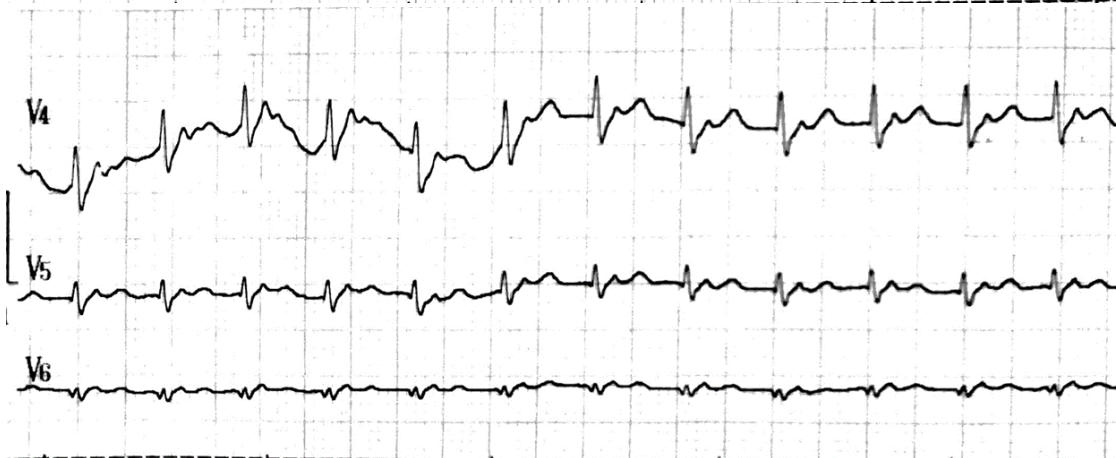

Supplement: Supplemental Digital Content [file medi-99-e22086-s001.pdf]

起始秒数 0.00 25.00 mm/s 10.00 mm/mV

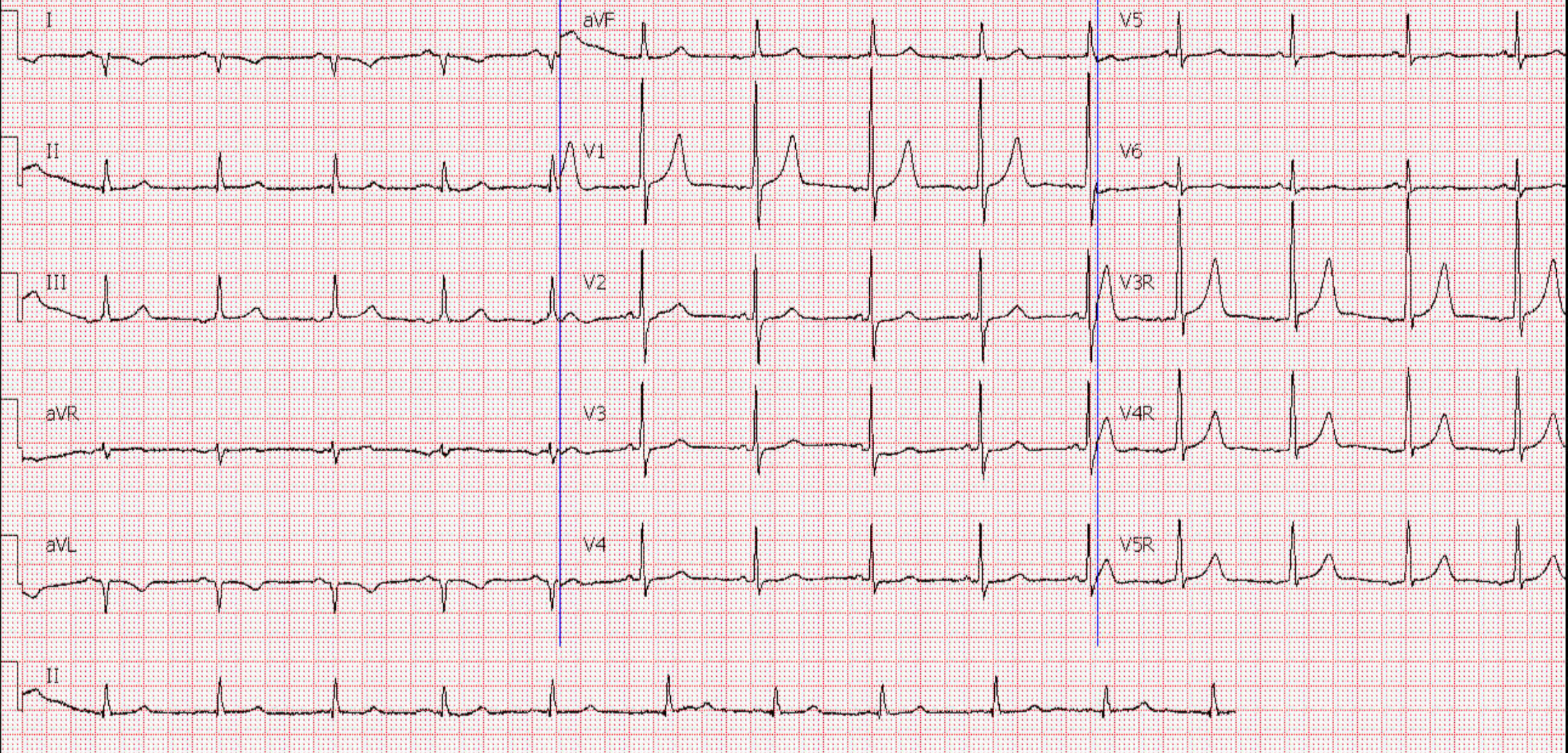

Supplement: Supplemental Digital Content [file medi-99-e22086-s002.pdf]
